# Benefits of topical natural ingredients in epidermal permeability barrier

**DOI:** 10.3389/fphys.2023.1275506

**Published:** 2024-01-04

**Authors:** Dongyun Lei, Dan Liu, Junling Zhang, Litao Zhang, Mao-Qiang Man

**Affiliations:** ^1^ Department of Dermatology, Tianjin Academy of Traditional Chinese Medicine Affiliated Hospital, Tianjin, China; ^2^ Dermatology Hospital, Southern Medical University, Guangzhou, China; ^3^ Dermatology Service, Veterans Affairs Medical Center San Francisco, Department of Dermatology, University of California San Francisco, San Francisco, CA, United States

**Keywords:** topical, natural ingredients, transepidermal water loss, epidermis, barrier, permeability

## Abstract

Because of the crucial role of epidermal permeability barrier in regulation of cutaneous and extracutaneous functions, great efforts have been made to identify and develop the regimens that can improve epidermal permeability barrier function. Studies have demonstrated that oral administration of natural ingredients can improve epidermal permeability barrier in various skin conditions, including inflammatory dermatoses and UV-irradiation. Moreover, topical applications of some natural ingredients can also accelerate the repair of epidermal permeability barrier after acute barrier disruption and lower transepidermal water loss in the intact skin. Natural ingredient-induced improvements in epidermal permeability barrier function can be attributable to upregulation of keratinocyte differentiation, lipid production, antioxidant, hyaluronic acid production, expression of aquaporin 3 and sodium-hydrogen exchanger 1. In this review, we summarize the benefits of topical natural ingredients in epidermal permeability barrier in normal skin with or without acute barrier disruption and the underlying mechanisms.

## 1 Introduction

Over the last decades, the regulatory role of epidermal permeability barrier in cutaneous and extracutaneous function has been well appreciated. Disruption of epidermal permeability barrier increases epidermal lipid and DNA syntheses (Feingold, 1991; Proksch et al., 1993). Similarly, barrier disruption increases release and synthesis of proinflammatory cytokines in the epidermis in addition to the increases in the density of Langerhans cells and mast cells in the dermis (Wood et al., 1992; Proksch et al., 1996; Proksch and Brasch, 1997; Wood et al., 1997; Lin et al., 2013). Moreover, compromised epidermal permeability barrier increases cutaneous inflammatory response to stimuli (Nishijima et al., 1997). Furthermore, disruption of epidermal permeability barrier function increases circulating levels of proinflammatory cytokines (Hu et al., 2017). Thus, prolonged, sustained cutaneous inflammation can induce chronic, systemic inflammation, which has been linked to the development of a variety of disorders, including type 2 diabetes, obesity, cardiovascular diseases and Alzheimer’s disease (Man and Elias, 2019). Conversely, improvement in epidermal permeability barrier function lowers expression levels of proinflammatory cytokines in both the skin and the circulation (Wood et al., 1994; Wood et al., 1996; Hu et al., 2017). In addition, defective epidermal permeability barrier function is associated with colonization of cutaneous *Staphylococcus aureus* (Na et al., 2012; Jinnestål et al., 2014). This line of evidence demonstrates the crucial role of competent epidermal permeability barrier in the maintenance of normal health condition.

Because of the regulatory role of epidermal permeability barrier in human health, approaches that can improve epidermal permeability barrier have been widely employed in the prevention and the treatment of some health conditions, such as eczematous dermatitis and psoriasis (Man et al., 2018; Man et al., 2019a; Man et al., 2019b; Park et al., 2021; Ní Chaoimh et al., 2023). Although oral administration of substances is suitable for improvement of epidermal permeability barrier in a larger surface area of the body, higher dose of active ingredients is usually required to achieve the benefits in epidermal function as compared to topical treatments. In addition, oral intake of substances can increase the risk of adverse events for the digestive system, kidney, and the liver. In contrast, topical applications of substances usually do not cause extracutaneous adverse events. Moreover, some body sites such as the hands and the face are often subject to external insults, resulting in disruption of epidermal permeability barrier. It is more appropriate to use topical products to improve epidermal permeability barrier function in these vulnerable body sites. Among a variety of ingredients that can improve epidermal permeability barrier, natural ingredients exhibit several advantages, such as lower cost, higher safety and availability, compared to synthetic chemicals. In this review, we brief the topical natural ingredients that benefit epidermal permeability barrier in normal skin with or without acute barrier disruption and the underlying mechanisms (Table 1).

**TABLE 1:** Topical natural ingredients that benefit epidermal permeability barrier.

Ingredients	Subjects	Treatments	Outcomes	Ref.
Acceleration of Barrier Recovery after Barrier Disruption				
Optimal Lipid Mixture	Aged mice	A mixture of cholesterol, fatty acid and ceramide at optimal ratio was applied to the skin after acute barrier disruption with tape-stripping	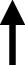 Barrier recovery	Zettersten et al. (1997)
	Young mice	A mixture of cholesterol, fatty acid and ceramide at optimal ratio was applied to the skin after acute barrier disruption with acetone	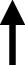 Barrier recovery	Mao-Qiang et al. (1995a), Yang et al. (1995)
Petrolatum	Young mice	Petrolatum was applied to the skin after acute barrier disruption with acetone	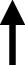 Barrier recovery	Mao-Qiang et al. (1995b)
Optimal Lipid Mixture	Aged Humans	A mixture of cholesterol, fatty acid and ceramide at optimal ratio was applied to the skin after acute barrier disruption with tape-stripping	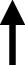 Barrier recovery	Zettersten et al. (1997)
Ceramides 1 and 3	Humans aged 20–30 years	Skin was treated with 17% SLS for 7 h, followed by applications of emollient twice daily for 28 days	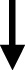 TEWL (both ceramide 1 and 3)	Huang and Chang (2008)
Ursolic acid (UA) and oleanolic acid (OA)	Mice	After acute barrier disruption with tape-stripping, either 0.01–0.1 mg/mL UA or 0.1–1.0 mg/mL ONA was applied to the tape-stripped area	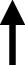 Barrier recovery (both UA and OA)	Lim et al. (2007)
Glycerol	Humans aged 24–35 years	After acute barrier disruption with tape-stripping, glycerol was applied to the tape-stripped area three times daily for 3 days	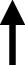 Barrier recovery	Fluhr et al. (1999)
	Humans aged 24–28 years	After barrier disruption with 10% SLS for 3 h, glycerol was applied to the SLS-treated area for 3 h	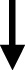 TEWL	Atrux-Tallau et al. (2010)
Canola Oil	Humans aged 22–57 years	After barrier disruption with 14% SLS for 7 h, the SLS-treated area was covered with canola oil for 17 h	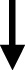 TEWL	Lodén & Andersson. (1996)
Sunflower Oil		After barrier disruption with 14% SLS for 7 h, the SLS-treated area was covered with sunflower oil for 17 h	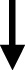 TEWL	
Sunflower Oil	Mice	Sunflower oil was applied to the skin after acute barrier disruption with tape-stripping	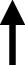 Barrier recovery	Darmstadt et al. (2002)
Extract of *Agrimonia pilosa Ledeb leaves*	Mice	Immediately after acute disruption of barrier with tape-stripping, herbal extract was applied to the tape-stripped area	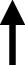 Barrier recovery	Nam et al. (2017)
Apigenin	Mice	Mice were treated topically with 0.1% apigenin twice daily for 9 days, followed by acute barrier disruption with tape-stripping	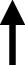 Barrier recovery	Hou et al. (2013)
Hesperidin	Mice	Mice were treated topically with 2% hesperidin twice daily for 6 days, followed by acute barrier disruption with tape-stripping	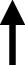 Barrier recovery	Hou et al. (2012)
Hesperidin	Aged mice	Aged mice were treated topically with 2% hesperidin twice daily for 9 days, followed by acute barrier disruption with tape-stripping	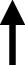 Barrier recovery	Man et al. (2015)
^b^Mixture of HA, glycerin, etc.	Humans with mean age of 40 years old	Single application of a mixture of hyaluronic acid 1%, glycerin 5%, and extract of *Centella asiatica* stem cells	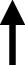 Barrier recovery	Milani and Sparavigna (2017)
Cannabis sativa L. extract	Humans aged 28–36 years	After acute barrier disruption with 1% SLS, the SLS-treated area was treated with hydrogel containing 0.5 or 1% of extract of Cannabis sativa L.	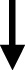 TEWL	Zagórska-Dziok et al. (2021)
^a^Ethanol Extract of an herbal mixture	Mice	1% of herbal extract was applied to mouse skin twice daily for 7 days, followed by tape-stripping	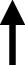 Barrier recovery	Man et al. (2011)
Cichorium intybus root extract	Humans aged 45–60 years	Skin was washed with 10% SLS, followed by topical application of 3% Cichorium intybus root extract twice daily for 28 days	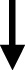 TEWL	Maia Campos et al. (2017)
Extract of comfrey root (Symphytii radix)	Young humans	Skin was occluded with 12% SLS for 6 h, followed by topical application of 1.12% comfrey root extract twice daily for 7 days	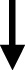 TEWL	Savić et al. (2015)
Attenuation of Barrier Disruption by External Stimuli				
Hesperidin	Mice	Mice were treated topically with 2% hesperidin and 0.05% clobetasol propionate twice daily for 9 days	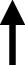 Barrier recovery	Man et al. (2014)
Rapeseed oil, Soy oil, Palm oil, petrolatum	Humans aged 20–38 years	Skin was treated with an oil for 10 min, followed by treatment with 0.5% SLS for 30 min. This procedure was repeated once daily for 4 days	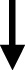 TEWL	Schliemann-Willers et al. (2002)
Chamomilla recutita extract	Humans	Addition of 0.1%–0.7% of Chamomilla recutita extract to dishwashing Liquids	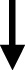 TEWL	Wasilewski et al. (2016)
Enhancing Permeability Barrier in Intact Skin				
Aqueous extract of Melissa officinalis (MO) leaves	Mice	Mice were treated topically with MO extract (5 mg/mL) every other day for 28 days	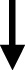 TEWL	Sipos et al. (2021)
Pinus halepensis bark extract	Mice	Mice were treated with the hydro alcoholic CMC gel containing the Pinus halepensis bark extract twice daily for 3 days	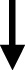 TEWL	Zoumpliou et al. (2014)
N-palmitoyl serinol	Mice	Mice were treated with topical 0.5% NPS twice daily for 1 week	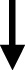 TEWL	Wen et al. (2021)
Aloe barbadensis	Humans aged -20–65 years	The face was treated with 10% of Aloe barbadensis cream (2 mg/cm^2^) for 15 days	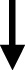 TEWL	Laneri et al. (2020)
hippophae rhamnoides fruit extract	Male humans aged 20–35 years	The cheek was treated with a cream containing hippophae rhamnoides fruit extract for 8 weeks	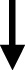 TEWL	Khan et al. (2011)
Emblica Officinalis Extract	Male humans aged 20–35 years	Skin was treated with 3% of Emblica Officinalis Extract for 8 weeks.	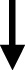 TEWL	Akhtar et al. (2012)
*Centella asiatica* extract	Humans aged 18–55 years	Skin was treated with *Centella asiatica* extract for 4 weeks	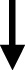 TEWL	Anggraeni et al. (2021)
Extract of Ashwagandha root (Withania somnifera)	Humans aged 18–60 years	Skin was treated with 8% standardized Ashwagandha root extract twice daily for 60 days	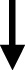 TEWL	Narra et al. (2023)
Extract of jade material	Humans aged 18–55 years	Extract of jade material was applied to the skin for 20 min three times per week for a total of 2 weeks	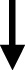 TEWL	Shu et al. (2023)
Melissa Officinalis Leaf Extract and Rosmarinic Acid	Humans aged 50–60 years	Skin was treated topically with either 0.05% of Melissa officinalis leaf extract or 0.1% rosmarinic acid twice daily for 4 weeks	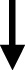 TEWL	Jung et al. (2022)
Stizolophus balsamita extract	Humans aged 35–61 years	Skin was treated topically with 3% of Stizolophus balsamita extract twice daily for 30 days	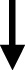 TEWL	Nawrot et al. (2021)
Coffee bean extract	Humans	Single application of coffee bean extract (0.2 mL of 100 μg/mL)	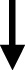 TEWL	Zofia et al. (2020)
kombucha berry leaf extracts	Humans	20 µL of kombucha berry leaf extracts (300 μg/mL) was applied in the skin	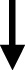 TEWL	Ziemlewska et al. (2022)
Ficus carica L. fruit extract	Humans aged 20–35 years	4% cream containing Ficus carica L. fruit extract was applied to the skin for 8 weeks	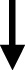 TEWL	Khan et al. (2014)
Aloe vera	Humans aged 15–50 years	Aloe vera cream was applied twice daily for 4 weeks	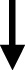 TEWL	Damayanti Umborowati et al. (2021)
Beeswax	Humans aged 20–30 years	10% of beeswax was applied daily for 28 days	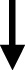 TEWL	Souza et al. (2017)
Mixture of shea butter, imperata cylindrica extract, coconut oil, Macadamia nut oil, Mangifera indica seed oil	Humans with mean age of 36.15 years	The skin was treated with the mixture daily for 2 weeks	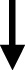 TEWL	Samadi et al. (2021)
^b^Mixture of HA, glycerin, etc.	Humans with mean age of 40 years old	Skin was treated with the mixture once	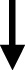 TEWL	Milani and Sparavigna (2017)

Abbreviations: TEWL, transepidermal water loss; SLS, sodium lauryl sulphate; HA, hyaluronic acid; 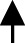: Accelerate recovery; 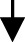: Decrease basal TEWL.

^a^ A mixture of Radix Paeonlae rubra, Cat Nut, Phelloden Dron, Rhizoma Alismatis, Angelica sinensis and Glabrous Greenbrier.

^b^ A mixture of HA, glycerin, and extract of *Centella asiatica* stem cells.

## 2 Natural ingredients that accelerate epidermal permeability barrier recovery after acute barrier disruption

The stratum corneum is the major site of epidermal permeability barrier, which is largely determined by the quality and quantity of a mixture of lipids, including ceramides, cholesterol and fatty acids (Man et al., 1996; Feingold and Elias, 2014). Of course, structural proteins are also the determinant for epidermal permeability barrier function (Haftek et al., 2021). Agents that stimulate keratinocyte differentiation and lipid production can improve epidermal permeability barrier homeostasis (Man et al., 2006; Schmuth et al., 2008). Correspondently, single topical application of an optimal lipid mixture (cholesterol, fatty acids and ceramide at a molar ratio of 3:1:1, any of these lipids can be 3) accelerates permeability barrier recovery by over 30% compared to the vehicle-treated controls in a murine model with acute barrier disruption with topical acetone treatment (Man et al., 1996). Similarly, single topical application of an optimal lipid mixture (cholesterol, ceramides, palmitate and linoleate = 3:1:1:1) accelerates barrier recovery 6 h after barrier disruption with tape-stripping in both aged mice and aged humans (Zettersten et al., 1997). Notably, free fatty acid-dominant lipid mixture delays permeability barrier recovery in aged mice although it accelerates recovery in young mice (Man et al., 1996; Zettersten et al., 1997). Such delayed barrier recovery is possibly due to the excessive content of medium and long chain fatty acids (C14-C18) in the epidermis because aged epidermis already has higher content of these fatty acids in comparison to young epidermis (Ghadially et al., 1995). It is known that excessive fatty acid delays permeability barrier recovery (Man et al., 1996). Moreover, topical application of an optimal lipid mixture (the molar ratio of cholesterol, ceramide, palmitate and linoleate = 4.3:2.3:1:1.08) improves barrier recovery in young mice following acute barrier disruption with either acetone or petrolatum ether or tape-stripping and some detergents, such as 15% N-laurosarcosine free acid and 10% dodecylbenzenesulphuric acid, but not with 10% sodium dodecyl sulphate nor 50% ammonium lauryl sulphosuccinate (Yang et al., 1995). The differential effects of optimal lipid mixture on skin treated with different detergents can be attributable to the extent of skin damage induced by these detergents. Either 10% sodium dodecyl sulphate or 50% ammonium lauryl sulphosuccinate requires 15 min to effectively disrupt the permeability barrier. In contrast, 10% dodecylbenzenesulphuric acid and 15% laurosarcosine free acid only require 5 and 6 min, respectively, to disrupt the permeability barrier (Yang et al., 1995). The longer the skin exposes to detergent, the more severe damage can be induced. Hence, optimal lipid mixture may not benefit barrier repair in sodium dodecyl sulphate- or ammonium lauryl sulphosuccinate-treated skin. Nonetheless, this line evidence indicates that the impact of optimal lipid mixture on epidermal permeability barrier homeostasis varies with age and the type of insults.

In our daily lives, our hands often contact a variety of detergents, which can disrupt epidermal permeability barrier and induce dermatitis. Following topical application of 17% sodium lauryl sulfate for 7 h, twice-daily applications of product containing either ceramide 1 or ceramide 3 for 2 days induce 12% reduction in transepidermal water loss rates (TEWL), an indicator of epidermal permeability barrier, while product containing both ceramide 1 and 3 lowers TEWL by 32% compared to the vehicle-treated site in humans (Huang and Chang, 2008). Taken together, this evidence demonstrates the benefit of stratum corneum lipids in epidermal permeability barrier homeostasis.

In addition to the stratum corneum lipids, topical plant oils also improve epidermal permeability barrier homeostasis after acute barrier disruption. For example, single topical application of sunflower oil accelerates barrier recovery by 31% and 16%, respectively, 1 and 5 h after barrier disruption with tape-stripping in mice whereas mustard oil, olive oil and soybean oil significantly delay barrier recovery (Darmstadt et al., 2002). The benefit of sunflower oil on permeability barrier homeostasis has also been demonstrated in humans with barrier disruption. Following challenges with 14% sodium lauryl sulfate, topical sunflower oil significantly lowers TEWL in human skin (Lodén and Andersson, 1996). However, neither borage oil nor shea butter lowers TEWL in human skin following the treatment with 14% sodium lauryl sulfate. Collectively, some plant oils accelerate barrier repair following acute barrier disruption.

Several studies have shown the benefits of herbal extract in epidermal permeability barrier homeostasis. In hairless mice, topical applications of 0.1% apigenin, a bioflavonoid found in chamomile tea and a variety of other plants, for 9 days markedly accelerate barrier recovery 4 h after barrier disruption with tape-stripping (Hou et al., 2013). Likewise, topical applications of 2% hesperidin, a dietary flavone glycoside mainly derived from citrus species, for 6 days accelerate barrier recovery by 37% and 23%, respectively, 2 and 4 h after barrier disruption with tape-stripping in young mice (Hou et al., 2012). Similar effects were also observed in aged mice (Man et al., 2015). Moreover, topical applications of extract of an herbal mixture (Radix Paeonlae rubra, Cat Nut, Phelloden Dron, Rhizoma Alismatis, Angelica sinensis and Glabrous Greenbrier) twice daily for 6 days significantly accelerate barrier recovery 2 and 4 h after barrier disruption with tape-stripping (Man et al., 2011). In addition, topical application of herbal extract improves barrier recovery when applied to barrier disrupted skin with tape-stripping. Following barrier disruption with tape-stripping, topical application of water extract of Agrimonia pilosa leaves does-dependently accelerates barrier recovery up to 30 h after application in mice (Nam et al., 2017). Topical cannabis sativa L. extract lowers TEWL by 20%–30% in mouse skin treated with 1% sodium lauryl sulfate (Zagórska-Dziok et al., 2021). Thus, a pile of evidence shows that topical extract of natural ingredients accelerates epidermal permeability barrier repair following acute barrier disruption in both the murine and human skin.

## 3 Natural ingredients that attenuate the disruption of epidermal permeability barrier by external stimuli

In addition to acceleration of permeability barrier recovery, attenuation of barrier abnormalities induced by insults is another approach to mitigate the negative impacts of external insults on epidermal permeability barrier. Some natural ingredients can diminish the abnormalities in epidermal permeability barrier induced by stimuli. For example, either topical or systemic administrations of glucocorticoids can delay epidermal permeability barrier recovery (Denda et al., 2000; Kao et al., 2003), whereas topical co-applications of 0.05% clobetasol and 2% hesperidin twice daily for 9 days override clobetasol-induced delay in barrier recovery 4 h after barrier disruption with tape-stripping, in addition to correction of clobetasol-induced elevation in skin surface pH (Man et al., 2014). Repeated exposure of the skin to detergents contributes to the development of eczema (Hamnerius et al., 2018; Jindal and Pandhi, 2020; Teo et al., 2023), likely resulting from the disruption of epidermal permeability barrier (Príborský et al., 1992; Okuda et al., 2002; Wolf and Parish, 2012). Pretreatment of the skin with some oils, such as palm fruit oil, soybean oil and rapeseed oil, largely prevents sodium lauryl sulfate-induced elevation in TEWL, evidenced by 22%–62% reductions in TEWL vs. untreated controls (Schliemann-Willers et al., 2002). Dishwashing liquid can irritate the skin and increase TEWL (Astner et al., 2006). Washing dishes with dishwashing liquid containing 0.1%–0.7% of Chamomilla recutita extract lowers TEWL by up to 40% in comparison to that without Chamomilla recutita extract (Wasilewski et al., 2016). Hence, topical applications of some natural ingredients can mitigate epidermal permeability barrier abnormalities induced by external stimuli.

## 4 Natural ingredients that enhance epidermal permeability barrier in intact skin

Compromised epidermal permeability barrier function increases cutaneous inflammatory response to stimuli and bacterial colonization (Nishijima et al., 1997; Scharschmidt et al., 2009; Na et al., 2012; Jinnestål et al., 2014). Therefore, enhancement of epidermal permeability barrier function can decrease cutaneous inflammation and infections when subjected to external insults. Several natural ingredients can enhance baseline epidermal permeability barrier function in the intact skin. In murine model, topical applications of an aqueous extract of Melissa officinalis leaves at a concentration of 5 mg/mL every other day induce a significant reduction in TEWL on day 12 (*p* < 0.05), with a further reduction on day 28 (*p* < 0.0001) (Sipos et al., 2021). Likewise, topical applications of pinus halepensis bark extract twice daily for 3 days markedly lower TEWL (*p* < 0.05) (Zoumpliou et al., 2014). Similarly, topical applications of 0.2% or 0.5% of N-palmitoyl serinol (NPS), a commensal bacterial metabolite, twice-daily for 1 week lower basal TEWL and accelerate barrier recovery by 20% (Wen et al., 2021). Moreover, the benefit of natural ingredients in epidermal permeability barrier function has also been demonstrated in humans. For example, topical applications of *centella asiatica* extract twice daily for 4 weeks decrease TEWL on the palm, with a comparable efficacy to ceramide (Anggraeni et al., 2021). However, other study did not show significant changes in TEWL on either the palm or the dorsal hand following topical applications of *centella asiatica* extract twice daily for 4 weeks (Damayanti Umborowati et al., 2021). In addition, topical extract of ashwagandha root twice daily for 60 days lowers TEWL by 40% (Narra et al., 2023). Topical treatments with product containing extract of hippophae rhmanoides berries also lower TEWL by 28% by 2 weeks and 50% reduction by 8 weeks in comparison to the placebo-treated skin (Khan et al., 2011). Impressively, a single topical application of coffee bean extract induces over 25% reduction in TEWL compared to untreated skin (Zofia et al., 2020). Regarding the influence of aloe vera on epidermal permeability barrier, the results are inconclusive although it is widely used in skin care products. One study showed that topical applications of cream containing 10% of aloe leaf extract decreased TEWL by 56% 27 h after application and by 65% 15 days after applications in humans (Laneri et al., 2020). Similarly, TEWL on the dorsal hand, but not on the palm, was decreased by topical applications of cream containing aloe extract twice daily for 4 weeks (Damayanti Umborowati et al., 2021). However, other studies did not show the benefit of topical aloe extract in epidermal permeability barrier function (Akhtar et al., 2011; Dal'Belo et al., 2006). In contrast, topical aloe extract increases epidermal permeability *in vitro* (Fox et al., 2015; Sharma et al., 2015). Thus, additional studies are needed to validate the benefit of topical aloe extract in epidermal permeability barrier. Apparently, some natural ingredients have long-lasting effects on epidermal permeability barrier function. For example, topical application of a product containing a mixture of coconut oil, Macadamia nut oil and Mangifera indica seed oil dramatically decreased TEWL within 1 hour after application (11.04 g/m^2^/hr vs. 9.6 g/m^2^/hr). A further decrease in TEWL was observed even 1 week after stopping application of the product (9.76 g/m^2^/hr vs. 7.88 g/m^2^/hr) (Samadi et al., 2021). Taken together, this line of evidence illustrates that topical applications of some natural ingredients enhance epidermal permeability barrier function in the intact skin.

## 5 Mechanisms by which natural ingredients improve epidermal permeability barrier

As aforementioned, epidermal permeability barrier is primarily determined by stratum corneum lipids and differentiation marker-related proteins. Natural ingredients improve epidermal permeability barrier mainly via direct or indirect regulation of epidermal lipid metabolism and keratinocyte differentiation.

### 5.1 Upregulation of epidermal lipid production

The critical role of three key stratum corneum lipids, including cholesterol ceramides, and fatty acids, in epidermal permeability barrier function has been well demonstrated in both humans and murine models (Feingold, 1991; Mao-Qiang et al., 1993; Mao-Qiang et al., 1995a; Yang et al., 1995; Zettersten et al., 1997; Huang and Chang, 2008). Topical application of stratum corneum lipid accelerates the formation of lamellar bilayers, a critical structure for epidermal permeability barrier, in the intercellular space of the stratum corneum (Mao-Qiang et al., 1995b; Yang et al., 1995; Zettersten et al., 1997), while some natural ingredients can upregulate expression levels of mRNA for enzymes required for epidermal lipid synthesis. The synthesis of three key barrier-related lipids requires their respective enzymes, 3-hydroxy-3-methyl glutaryl-CoA (HMGCoA), serine–palmitoyl transferase 1(SPT1), and fatty acid synthase (FAS), which all are rate-limiting enzymes in the early step of respective lipid synthesis pathway. Topical treatments of mouse skin with apigenin increase expression levels of mRNA for HMGCoA, SPT1, and FAS, accompanied by acceleration of lamellar body formation and secretion, a critical event to deliver lipids to the stratum corneum (Hou et al., 2013).

Chronologically-aged skin displays delayed permeability barrier recovery after barrier disruption (Wang et al., 2020), in part, due to reduction in epidermal lipid synthesis (Ghadially et al., 1995). Correspondingly, mRNA levels for all three key lipid synthetic enzymes are lower in the epidermis of the aged skin compared to that of the young skin (Man et al., 2015). Topical treatments with hesperidin significantly increase expression levels of mRNA for HMGCoA, SPT1 and FAS in the aged mouse epidermis (Man et al., 2015), suggesting that improvement in epidermal permeability barrier function by hesperidin is attributable, at least in part, to the upregulation of epidermal lipid production. Moreover, upregulation of epidermal lipid synthesis can also account for the improvement in epidermal permeability barrier by the extract of an herbal mixture, which increases expression levels of mRNA for SPT1 and fatty acid 2-hydroxylase by over 2-fold in the mouse epidermis, following twice-daily applications for 6 days (Man et al., 2011).

The signaling pathways involved in the increased lipid production by natural ingredients are not clear. But evidence suggests that some natural ingredient-induced improvement in epidermal permeability barrier is attributable to the upregulation of expression of peroxisome proliferator-activated receptors (PPAR). Previous studies showed that activation of PPARs improves epidermal permeability barrier via stimulation of epidermal lipid production (Schmuth et al., 2004; Man et al., 2006; Nazari et al., 2011). Hesperidin can increase expression levels of PPARγ *in vitro* and *in vivo* (Nazari et al., 2011; Ghorbani et al., 2012; Elshazly et al., 2018; Meng et al., 2018). In addition, either topical ursolic acid or oleanolic acid, which both accelerate permeability barrier in mice (Lim et al., 2007), increases PPARα expression in keratinocyte cultures (Lee et al., 2006; Lim et al., 2007). Thus, natural ingredient-induced increase in PPAR expression can contribute to the improved epidermal permeability barrier function.

The formation of lamellar bilayers requires lamellar bodies to deliver the lipids from the stratum granulosum to the stratum corneum, while maturation of lamellar bodies and their cargo content requires ATP-binding cassette A12 (ABCA12), a transmembrane glycosylceramide transporter (Mao-Qiang et al., 1995a; Lefévre et al., 2003; Smyth et al., 2008; Hotz et al., 2023). Humans with ABCA12 mutation exhibit defective epidermal permeability barrier (Lefévre et al., 2003; Hotz et al., 2023). Topical treatments with natural ingredients, such as hesperidin and extract of an herbal mixture, induce up to 8-fold increases in expression levels of ABCA12 mRNA (Man et al., 2011; Hou et al., 2012; Man et al., 2015). Additionally, maturation of lamellar bilayers in the stratum corneum is critical to form competent permeability barrier. Both secretory phospholipase and beta-glucocerebrosidase are required for maturation of lamellar bilayers (Holleran et al., 1994; Man et al., 1996; Hanley et al., 1997; Chan et al., 2011). Treatment of the skin with hesperidin increases the activity of epidermal β-glucocerebrosidase and accelerates the maturation of lamellar bilayers in mice (Man et al., 2014; Man et al., 2015). Taken together, increases in epidermal lipid production, lamellar body formation and maturation of lamellar bilayers account for the improved epidermal permeability barrier function by topical natural ingredients.

### 5.2 Stimulation of keratinocyte differentiation

During the terminal differentiation, the plasma membrane of keratinocytes is replaced by the cornified envelope, consisting of ∼80% loricrin, 8% small proline-rich proteins and 6% filaggrin, crosslinked by transglutaminase (Candi et al., 2005). The cornified envelope covalently binds to a monolayer of lipids, mainly ω-acylated-hydroxy-ceramides, forming the corneocyte-bound lipid envelope, which is an important structure for epidermal permeability barrier function (Elias et al., 2014). Therefore, regulation of differentiation marker-related proteins can affect epidermal permeability barrier function. Apigenin can upregulate expression levels of filaggrin in the mouse epidermis and expression levels of both filaggrin protein and mRNA in keratinocyte cultures (Hou et al., 2013). Similarly, hesperidin increases expression levels of filaggrin and loricrin proteins in both the aged and young mouse skin (Hou et al., 2012; Man et al., 2015). In parallel, mRNA levels for filaggrin, loricrin and involucrin are also increased in keratinocytes cultured with hesperidin (Man et al., 2015). Likewise, extract of royal jelly (a product of honeybees) at a concentration of 40 μm significantly increases expression levels of filaggrin mRNA and protein *in vitro* (Gu et al., 2017). Moreover, water extract of aloe vera increases filaggrin and involucrin expression in keratinocyte cultures (Razia et al., 2021). Additionally, agrimonia pilosa leaf extract increases transglutaminase activity by almost 100% over the controls in keratinocyte cultures (Nam et al., 2017). Hence, stimulation of keratinocyte differentiation is another mechanism accounting for the improvement in epidermal permeability barrier function by topical natural ingredients.

### 5.3 Anti-oxidative stress

Oxidative stress has been linked to compromised epidermal permeability barrier in some cutaneous conditions such as dermatitis and UV irradiation (Rojo de la Vega et al., 2017; Bertino et al., 2020; Yang et al., 2022). Accordingly, administrations of antioxidants improve epidermal permeability barrier function (Kuriyama et al., 2002; Masaki, 2010; Campos et al., 2014). Some natural ingredients exhibit potent antioxidant capacity. Previous study showed that extract of cannabis sativa L. scavenged 40% of the 1,1-diphenyl-2-picrylhydrazyl radical and increased superoxide dismutase activity by ≈ 70%, in addition to a reduction in intracellular reactive oxygen species in keratinocyte cultures (Zagórska-Dziok et al., 2021). Similarly, coffee bean extract increases superoxide dismutase activity by 50% *in vitro* (Zofia et al., 2020). Aqueous extract of agrimonia Pilosa also exhibits radical-scavenging property with IC50 value of as low as 5.6 μg/mL (Zhu et al., 2009). Reduction in intracellular reactive oxygen species was also observed in keratinocyte cultures following the treatment with extract of kombucha berry leaves (Ziemlewska et al., 2022). Thus, antioxidant of the natural ingredients is an additional mechanism accounting for the improvement in epidermal permeability barrier function.

### 5.4 Others

Several other mechanisms can also contribute to the natural ingredient-induced improvement in epidermal permeability barrier function. First, the epidermis expresses antimicrobial peptides, including cathelicidin-related peptide, which is packaged within and secreted by lamellar bodies (Oren et al., 2003; Braff et al., 2005). Cathelicidin-related peptide is required for and regulated by epidermal permeability barrier function (Aberg et al., 2008). Topical applications of either apigenin or hesperidin or extract of an herbal mixture markedly increased expression levels of cathelicidin-related peptide in the mouse epidermis (Man et al., 2011; Hou et al., 2012; Hou et al., 2013). Second, maturation of lamellar bilayers, a critical structure for epidermal permeability barrier, requires enzymatic processing of the lipids in the stratum corneum (Holleran et al., 1994; Mao-Qiang et al., 1995b; Chan et al., 2011). The optimal pH for those lipid processing enzymes is ≈ 5 (Takagi et al., 1999). Both sodium/hydrogen exchanger 1 (NHE1) and acidic secretory phospholipase A2 are the key regulator of the stratum corneum pH. Either inhibition of sPLA2-I or NHE1 deficiency delays epidermal permeability barrier recovery following acute barrier disruption (Mao-Qiang et al., 1993; Mao-Qiang et al., 1995a; Mao-Qiang et al., 1996; Fluhr et al., 2001; Behne et al., 2002; Fluhr et al., 2004). Topical hesperidin increases expression levels of epidermal NHE1 and sPLA2 (sPLA2g2f) mRNA by over 60% in mice (Man et al., 2015). While rosmarinic acid activates NHE1, Melissa officinalis leaf extract upregulates expression levels of NHE1 protein and mRNA in keratinocyte cultures (Jung et al., 2022). Third, hyaluronic acid can stimulate keratinocyte differentiation and lipid production, resulting in improvement in epidermal permeability barrier homeostasis (Bourguignon et al., 2006; Bourguignon et al., 2013). Extract of hippophae rhamnoides at a concentration of 10 μg/mL significantly increases expression levels of hyaluronan synthase mRNA and protein in keratinocyte cultures (Yao et al., 2021). Aloe vera extract also increases hyaluronic acid production and hyaluronan synthase expression in keratinocyte cultures (Razia et al., 2021). Moreover, aquaporin-3 (AQP3) is a water-, glycerol-, and hydrogen peroxide-transporter expressed in the epidermis. AQP3 deficiency delays permeability barrier recovery (Hara et al., 2002), while overexpression of AQP3 accelerates permeability barrier recovery (Qin et al., 2011). Extract of either hippophae rhamnoides or aloe vera or royal jelly can significantly increase aquaporin 3 expression in keratinocyte cultures (Gu et al., 2017; Razia et al., 2021; Yao et al., 2021). Additionally, transient receptor potential ion channel 3 (TRPV3) also regulates epidermal permeability barrier. Mice with TRPV3 deficiency display defective epidermal permeability barrier (Cheng et al., 2010). Treatment of keratinocytes with extract of agrimonia pilosa leaves induces over 4-fold increases in TRPV3 activity (Nam et al., 2017). Taken together, this bulk of evidence demonstrates that natural ingredients improve epidermal permeability barrier function via multiple mechanisms.

## 6 Potential clinical applications of natural ingredients in the management of skin conditions with compromised permeability barrier function

**FIGURE 1:**
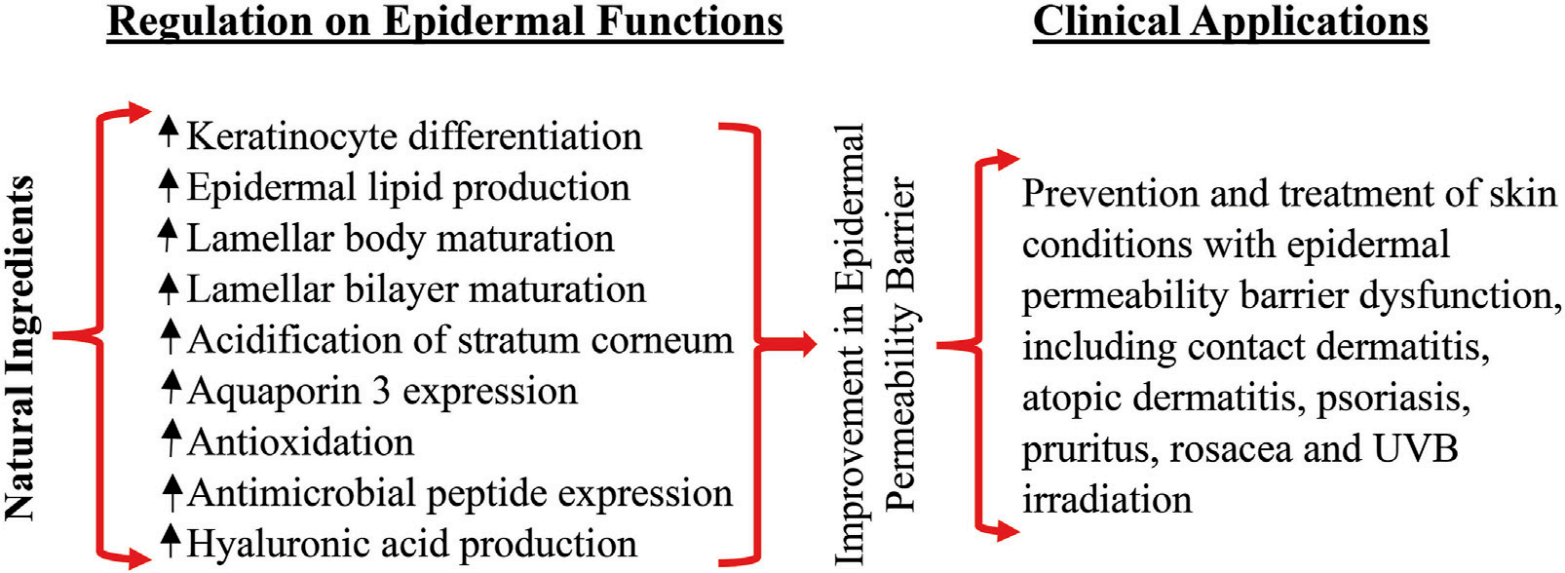
Benefits of topical natural ingredients on epidermal permeability barrier and clinical applications.

Because of the crucial role of epidermal permeability dysfunction in some dermatoses (Elias and Schmuth, 2009; Ye et al., 2014; Hatano and Elias, 2023), strategy that improve epidermal permeability barrier has been deployed in the treatment and prevention of skin disorders with defective permeability barrier. Previous studies showed that topical applications of emollient containing petrolatum, glycerol and sunflower seed oil, prevent the relapse of psoriasis (Man et al., 2019a). Similarly, topical applications of product containing Spa water and urea prevent the relapse of psoriasis following the treatment with glucocorticoids (Seité et al., 2009). Atopic dermatitis is another common skin disorder with defective epidermal permeability barrier. Topical applications of sunflower seed oil, which is known to improve epidermal permeability barrier (Darmstadt et al., 2002), reduce the risk for the development of atopic dermatitis in infants with high risk of atopic dermatitis (Simpson et al., 2014). Moreover, topical applications of a mixture of cholesterol, fatty acids and ceramides at a molar ratio of 1:1:3 for 28 days improve Severity Scoring for Atopic Dermatitis, pruritus and sleep habit scores in patients with atopic dermatitis, with comparable efficacy to fluticasone (Sugarman and Parish, 2009). Furthermore, topical applications of either beeswax or honey markedly lower TEWL in humans (Pavlačková et al., 2020). In parallel, topical treatments of either atopic dermatitis or psoriasis with honey mixture (honey, beeswax and olive oil at a ratio of 1:1:1) improve disease severity and reduce the dose of glucocorticoids (Al-Waili, 2003). Likewise, topical applications of a mixture of honey, beeswax and olive oil at a ratio of 4:1:2 improve diaper dermatitis (El Sakka et al., 2013). Taken together, the bulk of evidence indicates topical natural ingredients can improve epidermal permeability barrier function and alleviate skin disorders with defective epidermal permeability barrier. The benefits of topical natural ingredients on epidermal permeability barrier and possible clinical application are illustrated in Figure 1.

In summary, a variety of natural ingredients used alone or in combination can improve epidermal permeability barrier function in normal intact skin or following acute barrier disruption. The underlying mechanisms by which natural ingredients improve epidermal permeability barrier function include stimulation of keratinocyte differentiation, lipid production, antioxidant, activation of TRPV3, and upregulation of AQP3 and hyaluronic acid production. Topical applications of natural ingredients can accelerate the repair of epidermal permeability barrier after acute damage and enhance the permeability barrier to prevent the penetration of harmful substances into the skin, which is particularly important for some body sites such as the hands that are vulnerable to damage and exposure to harmful substances. However, available evidence cannot draw a conclusion which ingredient is superior to the others in term of efficacy because of lacking the data of side-by-side comparison.
